# Overview of antimicrobial resistance mitigation efforts in Benin 2024

**DOI:** 10.4102/jphia.v16i1.1332

**Published:** 2025-07-26

**Authors:** Alain K. Aissi, Tokpanou E.C. Koudjo, Filémon T. Tokponnon, Al Fattah Onifade, Akpeedje A.C. Wadagni, Géraud S.R. Padonou, Hervé A. Gbegnide, Léopold A. Azakpa, Adrien M. Hodonou, Roch C. Johnson, Francis M. Dossou

**Affiliations:** 1 Department of Training and Research in Health, Center for Entomological Research of Cotonou, Ministry of Health, Cotonou, Benin; 2 Laboratory of Hygiene, Sanitation, Toxicology, and Environmental Health, Interfaculty Center for Training and Research in Environment for Sustainable Development, University of Abomey-Calavi, Abomey-Calavi, Benin; 3 Beninese Society of Hospital Hygiene and Infection Prevention and Control, Abomey-Calavi, Benin; 4 General Directorate of Hospital Medicine and Diagnostic Explorations, Ministry of Health, Cotonou, Benin; 5 Polytechnic School of Abomey-Calavi, University of Abomey-Calavi, Abomey-Calavi, Benin; 6 World Health Organization, Cotonou, Benin; 7 National Council for the Fight Against HIV/AIDS, Tuberculosis, and Malaria, Ministry of Health, Cotonou, Benin; 8 Department of Training and Research in Health, Ministry of Health, Cotonou, Benin; 9 Faculty of Health Sciences, University of Abomey-Calavi, Abomey-Calavi, Benin; 10 Faculty of Medicine, University of Parakou, Parakou, Benin

**Keywords:** antimicrobial resistance, infection prevention and control, One Health, Benin, surveillance, antimicrobial stewardship, National Action Plan

## Abstract

**Background:**

Antimicrobial resistance (AMR) is a significant global public health threat that undermines sustainable development. In Benin, high AMR rates to common antibiotics, including last-resort treatments, exacerbate infection outcomes and healthcare costs. Inappropriate antibiotic use worsens this challenge. To address these issues, Benin implemented its National Multisectoral AMR Action Plan 2019–2024.

**Aim:**

This study aims to assess the implementation of Benin’s National AMR Action Plan 2019–2024 and identify critical gaps for future updates.

**Setting:**

The evaluation covered six of Benin’s 12 departments, engaging stakeholders from human, animal and environmental health sectors.

**Methods:**

A retrospective cross-sectional evaluation was performed from 05 August 2024 to 30 September 2024. Data were collected through a documentary review, standardised questionnaires, semi-structured interviews and group discussions. An executed scoring grid and a Strengths, Weaknesses, Opportunities and Threats analysis were used.

**Results:**

Of the 224 planned activities, 40.18% were not initiated, 31.25% had started, 19.64% were partially executed, 5.36% were nearly completed and only 3.57% were fully implemented. Critical gaps included the lack of a fully operational multisectoral coordination group, limited financial resources, inadequate surveillance systems, insufficient communication and education initiatives and the absence of infection prevention and control (IPC) programmes.

**Conclusion:**

Despite progress in regulatory frameworks, gaps in coordination, resources, surveillance and IPC hinder effective AMR control in Benin. Strengthening governance, communication and addressing other identified gaps are critical for future success.

**Contribution:**

This evaluation provides evidence-based recommendations to update Benin’s AMR strategy within a ‘One Health’ framework.

## Introduction

Antimicrobial resistance (AMR) is one of the most significant global public health threats, impacting sustainable development by affecting social well-being, poverty reduction, food security, the environment and economic growth^1^. In 2019, more than 4.95 million deaths were associated with AMR^2,3^, a drastic increase from the estimated 700 000 deaths in 2015^4^. If concrete actions are not implemented, AMR could directly cause 39.1 million cumulative deaths worldwide between 2025 and 2050^2^, equating to more than three deaths per minute, a 75% increase over 25 years^5^. While no country or individual is spared, West Africa is among the most affected regions, with a potentially disastrous economic impact^6,7^. Animals are equally affected by AMR as they face similar infection risks as humans. Consequently, this often necessitates the use of antimicrobials in veterinary care and agriculture^8^.

In Benin, a 2012 national survey highlighted significant resistance among Gram-negative bacteria to ampicillin (74% – 100%), amoxicillin–clavulanate (56% – 100%), ceftazidime (55% – 100%) and tetracycline (75% – 95%)^9^. In the same study, 52.5% of *Staphylococcus aureus* isolates were resistant to methicillin (MRSA), and 67.5% of enterococci were resistant to vancomycin^9^. Recent studies confirm the alarming trend of resistance to commonly used antibiotics, including those reserved for last-resort treatments^10,11^. Data indicate the emergence of extended-spectrum beta-lactamases (ESBLs)^9,10^ and carbapenem-resistant organisms (bla OXA-48, blaNDM, blaVIM producers) in surgical site infections^12^. This situation is exacerbated by inappropriate antibiotic use, including frequent self-medication, over-the-counter sales and a lack of clear guidelines, impacting livelihoods dependent on antibiotics^9,10,12^. The complexity of AMR development and spread requires coordinated control measures across multiple interdependent sectors and subsectors, in a One Health approach, integrating human, animal and environmental health, as well as plant production and food security^1,13,14^. To align with the Global Action Plan on AMR, Benin developed its National Multisectoral AMR Action Plan for 2019–2024^15^. This followed a 2017 Joint External Evaluation of International Health Regulations (IHR)^16^, which revealed limited capacity in AMR detection, reporting and monitoring, as well as weak infection prevention and control (IPC) and a lack of antimicrobial management plans. These issues were consistent with findings from a 2014 Veterinary Services Performance Evaluation, a gap analysis against the standards of the World Organisation for Animal Health (WOAH) in 2014^17^.Benin’s National AMR Action Plan is structured into five strategic axes, divided into 12 domains, 27 interventions and 65 actions, ultimately comprising 224 activities across human, animal and environmental health sectors. It has a budget of $11 666 000^15^, with a detailed breakdown available in the supplementary materials (Online Appendix 1). As the implementation period concludes, an evaluation is underway to identify gaps and inform future updates. Thus, this study aims to evaluate the implementation of Benin’s National Multisectoral AMR Action Plan 2019–2024, identify key gaps and provide recommendations to inform future updates and strengthen AMR mitigation efforts in the country. Our findings reveal that only 3.6% of planned activities were fully implemented, with critical gaps in multisectoral coordination, financial resources, surveillance and IPC. These results highlight the urgent need to reinforce governance, resource allocation and cross-sectoral collaboration to effectively address AMR in Benin.

## Research methods and design

### Study type and period

This is a retrospective cross-sectional evaluation with descriptive and analytical objectives. It was conducted from 05 August 2024 to 30 September 2024 by a multidisciplinary technical team established by the General Directorate of Hospital Medicine and Diagnostic Explorations (DGMHED) with the support of an independent consultant and the World Health Organization (WHO) office in Benin. This multidisciplinary team was composed of experts in human health, animal health, environment, epidemiology, microbiology and health systems management, ensuring a holistic and integrated approach to the evaluation. The study involved a documentary review and a survey that included direct observations, interviews and consultations with multisectoral stakeholders.

### Data collection

#### Documentary review

The review covered a variety of documents related to the AMR issue in Benin: legislative and regulatory texts; Tripartite AMR Country Self-Assessment Survey (TrACSS) reports on national progress in AMR control, joint evaluation reports in 2024 using Food and Agriculture Organization (FAO) of the United Nations Progressive Management Pathway for Antimicrobial Resistance (FAO-PMP-AMR) and FAO Assessment Tool for Laboratories and AMR Surveillance Systems (FAO-ATLASS), the 2023 joint external evaluation report of the IHR and the 2024 review workshop report on AMR quantitative data organised by the Ministry of Health.

#### Surveys, observations and stakeholder consultations

Although the AMR plan is national in scope and its implementation concerns all 12 departments of the country, the survey took place in six departments (Atlantique, Littoral, Ouémé, Plateau, Collines and Borgou) selected to offer a representative sample of the geographic, demographic, agricultural and health diversity of Benin while considering practical, logistical and budgetary constraints. The choice was also motivated by the desire to obtain a balanced representation of the situations and challenges encountered in the country. These six departments are not limited to certain geographic areas or specific characteristics but were chosen for their diversity and commitment to implementing the AMR plan.

In total, the study sample consisted of 305 actors representing the main categories of stakeholders identified in the 2019–2024 AMR plan. Among them, 209 people were surveyed using standardised questionnaires, 38 people participated in semi-structured individual interviews and 58 people participated in group discussions, allowing for a more in-depth exploration of the information. The combination of these three data collection methods made it possible to triangulate the information and obtain a more complete view of the situation.

The inclusion criteria for these participants were their role and level of involvement in the implementation of the AMR plan, their institutional affiliation (membership in the human, animal and environmental health sectors), their technical expertise and their direct or indirect contribution to AMR control activities. These criteria ensured that the participants possessed in-depth knowledge of the context and challenges related to AMR in Benin. Approximately 10% of the interviews were conducted by telephone and videoconference, particularly for resource persons who were not physically present but agreed to contribute to the collection of qualitative data.

The respondents from the human health sector were from the three levels of the healthcare pyramid, including the DGMHED; the National Agency for Primary Health Care; the Beninese Agency for Medicines and Other Health Products; the National Agency for Quality Control of Health Products; the National Council for the Fight Against HIV/AIDS, Tuberculosis, Malaria, sexually transmitted infections (STIs), hepatitis and epidemics; six Regional Health Directorates; six Health Zone Coordination Offices; seven district hospitals; four university teaching hospitals; 12 pharmacies and two pharmaceutical wholesalers.

Respondents from the animal health sector included the Directorate of Livestock; the Directorate of Plant Production; the Directorate of Fisheries and Aquaculture; the Beninese Agency for Food Safety; six Regional Directorates of Agriculture, Livestock and Fisheries; the Association of Private Veterinary Doctors and one importer of veterinary medicines and phytosanitary products, as well as livestock farmers and agricultural producers.

Respondents from the environmental health sector were affiliated with the Ministry of Living Environment, Transport and Sustainable Development, specifically the General Directorate of Environment and Climate and the Laboratory for Environmental Study and Monitoring. Additionally, other ministries were involved, including the Ministry of Higher Education and Scientific Research; the Ministry of Primary Education; five research laboratories; a consumer association and technical and financial partners such as the WHO, FAO and WOAH.

#### Data processing and analysis

The data were entered into Kobotoolbox or directly into Microsoft^®^ Excel 2019, depending on the tool used. They were verified, summarised and classified to highlight the descriptive elements of the current situation using tables and graphs.

To assess the level of implementation of activities, actions, interventions and strategies by axis, a scoring grid inspired by WHO assessment tools^18^ was used (Table 1). The criteria for assigning scores were defined using the following execution levels: Not started (0%) = the activity has not commenced in any sector; initiated (25%) = the activity has been initiated in at least one sector, or preparatory steps have begun; in progress (50%) = the activity is underway, with approximately half of the expected results achieved; nearly completed (75%) = the activity is almost finished, with most results achieved; fully completed (100%) = the activity is fully implemented across all relevant sectors. Note that ‘sector’ refers to the human health sector, animal health sector and environmental sector. For example, an activity was considered ‘in progress’ (50%) if data collection was actively underway but not yet completed in the relevant sectors. An activity was considered ‘nearly completed’ (75%) if a draft report had been prepared and was awaiting finalisation and dissemination. An activity was considered ‘fully completed’ (100%) if all planned steps had been carried out, the final report had been disseminated, and the results were being used to inform decision-making. The process of assigning scores involved expert judgement, and efforts were made to ensure consistency among all evaluators through detailed training and calibration exercises. Clear definitions and examples for each execution level were provided to the evaluators during training. After assigning execution levels for activities in the Excel template, averages were calculated successively for each action, intervention, strategy and axis.

**TABLE 1:** Criteria for scoring levels of activity implementation.

Execution level	Score (%)	Interpretation
Not started	0	The activity has not started in any sector (human health, animal health or environmental health).
Initiated	25	The activity has been initiated in at least one sector. For multisectoral activities, preparatory steps (e.g. drafting terms of reference + resource mobilisation) have begun.
In progress	50	The activity is underway, with approximately half of the expected results achieved in at least one sector. For multisectoral activities, at least one sector has made significant progress.
Nearly completed	75	The activity is almost finished with most results achieved. For multisectoral activities, at least two sectors have reached advanced stages of implementation, or one sector has completed implementation.
Fully implemented	100	The activity is fully implemented. For multisectoral activities, all sectors involved have completed implementation effectively.

To aid in quickly visualising and interpreting the implementation levels, colour-coded graphs were automatically generated, where red indicated a low level of implementation (not started or initiated). As the score increases, the colour transitions to orange and then green. Orange represents an intermediate level (in progress), while green indicates a high level of implementation (nearly or fully completed). Intermediate shades of these colours represent a gradation correlated with the execution level.

The strengths, weaknesses, opportunities and threats (SWOT) related to the national AMR action plan implementation were identified using a matrix.

### Ethical considerations

This study did not involve biomedical interventions or experiments on human or animal subjects. However, as it included surveys with stakeholders, permission from the Ministry of Health was obtained prior to initiation through the General Directorate of Hospital Medicine and Diagnostic Explorations (DGMHED) and the National Council of Hospital Medicine (NCHM). Informed consent was obtained from all participants prior to data collection. They were informed of the purpose of the study, the voluntary nature of their participation and their right to withdraw at any time without consequences. Confidentiality was ensured by the anonymisation of responses and the secure storage of all data collected. No personally identifiable information was recorded or disclosed. All procedures performed were in accordance with the ethical standards and the 1964 Helsinki Declaration and its later amendments.

## Results

### Antimicrobial resistance National Action Plan implementation level

**FIGURE 1:**
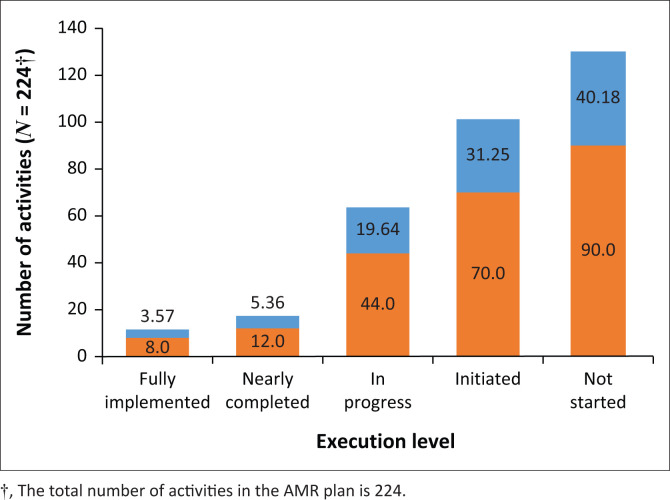
Distribution of activities by implementation level in Benin’s National Multisectoral Antimicrobial Resistance Action Plan 2019–2024 as of 31 August 2024.

Figure 1 presents the implementation status of the 224 activities described in the National Action Plan to Combat AMR. These activities cover five strategic axes (knowledge promotion, surveillance, IPC, antimicrobial management and coordination) and are detailed in a supplementary document (Online Appendix 1). Of these activities, 90 (40.18%) have not been initiated and remain unexecuted. In total, 70 activities (31.25%) have either started in at least one sector or are in the preparatory phase, such as drafting terms of reference. Meanwhile, 44 activities (19.64%) are partially executed, with at least half of the expected results achieved in at least one sector. Additionally, 12 activities (5.36%) are nearly completed, and only 8 activities (3.57%) have been fully executed (Figure 1).

**FIGURE 2:**
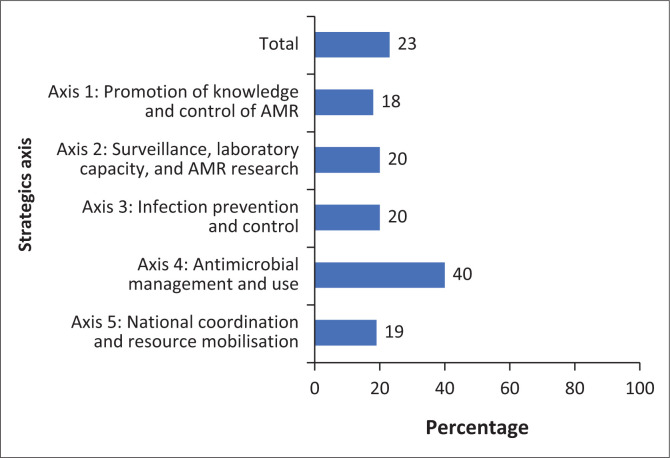
Implementation level of activities by strategic axis of Benin’s National Multisectoral Antimicrobial Resistance Action Plan 2019–2024.

**FIGURE 3:**
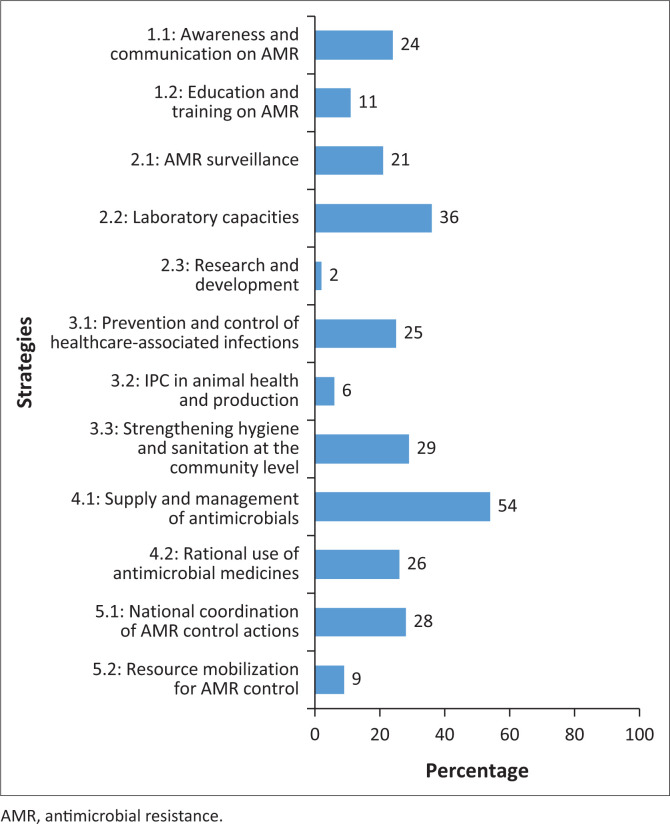
Implementation level of activities by strategic domain of Benin’s National Multisectoral Antimicrobial Resistance Action Plan 2019–2024 as of 31 August 2024.

Figure 2 presents a summary of implementation scores by strategic axis as of 31 August 2024 within the framework of the overall evaluation of the National Action Plan to Combat AMR. The overall implementation level stands at 23%, highlighting significant disparities between strategic domains (Figure 3). For example, Axis 4, related to antimicrobial use and management, shows the highest performance with an overall implementation rate of 40%. Within this axis, the specific domain of antimicrobial supply and management achieves an execution rate of 54% (Figure 3).

The strategic domains with the lowest execution rates are, in ascending order: research and development (2%), IPC in animal health and production (6%), resource mobilisation for AMR control (9%) and education and training (11%). The implementation score for laboratory capacity activities is 36%.

The supplementary document (Online Appendix 1) provides detailed scores for activities, along with averages for actions, interventions, strategies and axes.

### Strengths, weaknesses, opportunities and threats analysis by strategic axis of the antimicrobial resistance national action plan

To provide a comprehensive overview of Benin’s capacity to address AMR, we conducted a SWOT analysis. This framework allows for a structured assessment of the internal and external factors influencing the country’s AMR mitigation efforts. Table 2 presents a synthesis of the SWOT as of 31 August 2024. The SWOT analysis is organised by strategic axis of the National Action Plan on AMR 2019–2024, offering insights into the key areas requiring attention and strategic action.

**TABLE 2:** Strengths, weaknesses, opportunities and threats related to the fight against antimicrobial resistance in Benin as of 31 August 2024.

Items	Key elements
Strengths	Existence of AMR focal points in the human, animal and environmental health sectors; Annual awareness campaigns during World Antibiotic Awareness Week; Organisation of several training, workshops on AMR and related topics, such as IPC, for human and animal health professionals; Integration of AMR into the training curricula of certain human and animal health professionals; Availability of certain public and private laboratories capable of generating AMR data in human and animal health; Adoption of a 5-year IPC plan for healthcare-associated infections; Implementation of nosocomial infection control committees in all hospitals; Organisation of multiple training sessions for hospital IPC committees; Development of a national protocol for antibiotic prophylaxis and empirical antibiotic therapy; Existence of national structures responsible for regulating and approving antimicrobials for human and animal health, as well as pesticides; Availability of updated national lists of essential medicines, including antimicrobials for human, animal and plant health; Existence of a national laboratory for quality control of medicines and health products, including veterinary medicines; Organisation of crackdowns on informal medicine markets in the human and animal health sectors; Existence of a circular prohibiting the dispensing of antibiotics without a medical prescription duly prescribed by a doctor.
Weaknesses	Absence of a formalised multisectoral coordination group (MCG) with a clear mandate; Insufficient financial resources for the implementation of the national action plan; Weak multisectoral collaboration; Inadequate monitoring and evaluation of AMR control indicators; Absence of a comprehensive communication plan based on a thorough analysis of AMR stakeholders; Weak ownership of the national AMR action plan by the main actors of the implementation structures; Weak involvement of the aquatic health, agriculture and food safety sectors in AMR awareness and training; Absence of AMR teaching in schools and certain university courses, such as plant and food production. Absence of a nationally coordinated surveillance system; Poor monitoring of antimicrobial consumption, particularly in animal health and agriculture; Absence of a surveillance system for AMU in the human, animal and plant health sectors; Limited national coverage of bacteriology laboratories; Limited research initiative on AMR in a multidisciplinary and multisectoral approach; Absence of a clearly defined national IPC programme in human health; Absence of national IPC guidelines based on evidence and adapted to the local context; Limited integration of water, sanitation and hygiene (WASH) in human, animal and agricultural health facilities, as well as in community settings such as schools and markets; Insufficient resources for hygiene and sanitation activities aimed at preventing healthcare-associated infections and combating AMR; Weak mechanisms for detecting counterfeit antimicrobials; Insufficient number of qualified professionals for antimicrobial prescription and infection monitoring in terrestrial and aquatic animals; Absence of national guidelines on the use of antimicrobials in animal health; Limited implementation of the electronic logistics management system to facilitate stock management and reduce the risk of antimicrobial shortages or expiration.
Opportunities	Commitment from international partners to support the fight against AMR; Drafting of an interministerial decree to formalise the national One Health platform; Creation of scientific societies for infectiology and IPC; Expansion of the One Health approach; Benin’s participation in various international and sub-regional meetings dealing with AMR and IPC; Creation of degree or certification training programmes to improve the number and quality of human resources involved in the fight against AMR.
Threats	Crises reducing funding opportunities from partners; Decreasing effectiveness of antimicrobials; Influence of stakeholders in the counterfeit medicine trade; Lack of enthusiasm for information-sharing among AMR control stakeholders.

AMR, antimicrobial resistance; AMU, antimicrobial use; IPC, infection prevention and control.

## Discussion

This evaluation of the implementation of Benin’s National AMR Action Plan (2019–2024) revealed a mixed picture of progress. While the plan has established a foundation for addressing AMR, as evidenced by the presence of AMR focal points and some regulatory frameworks, overall implementation remains limited. Key challenges include a lack of a fully functional multisectoral coordination group (MCG), inadequate resource mobilisation and insufficient surveillance systems, resulting in only 23% of planned activities being fully or nearly completed. These findings highlight the urgent need for targeted interventions to strengthen Benin’s response to AMR.

### Coordination and governance in antimicrobial resistance control

The lack of a formalised MCG with a clear mandate remains a significant impediment to Benin’s AMR response. While AMR focal points exist in the human, animal and environmental health sectors, the absence of a well-defined governance mechanism hinders unified national efforts and makes it difficult to mobilise sufficient financial resources for sustained implementation of the national action plan. Similar challenges have been reported in other low- and middle-income countries, where weak coordination has been identified as a major barrier to effective AMR control^19,20^. To address this, Benin should prioritise the formal establishment of a fully functional MCG with clearly defined roles, responsibilities and reporting mechanisms, as well as dedicated funding streams to support its activities. The drafting of an interministerial decree to formalise the national One Health platform presents a valuable opportunity to operationalise multisectoral AMR governance.

A functional MCG would enhance the monitoring and evaluation of AMR control indicators while ensuring the capitalisation of certain actions taken by projects or programmes combating pandemics (coronavirus disease 2019 [COVID-19], HIV/AIDS, malaria and tuberculosis). For instance, some interventions in response to COVID-19 have positively influenced the adoption of better anti-infective measures^21,22^. However, the COVID-19 crisis also disrupted the implementation schedule of several AMR plan activities between 2020 and 2022^23,24^.

### Antimicrobial resistance communication and education

The absence of a comprehensive communication plan based on a thorough analysis of AMR stakeholders compromises the effectiveness of awareness efforts in Benin. Although some sporadic awareness activities have been conducted, particularly during World Antibiotic Awareness Week^25,26^, they often fail to reach certain key stakeholders, especially in the animal health, agriculture and food production sectors^26^. The organisers of these campaigns are generally human health professionals, and the involvement of actors from other sectors often appears as a mere symbolic representation to satisfy the One Health approach. This limited scope contributes to a poor understanding of AMR issues, including among key stakeholders responsible for implementing AMR plan activities^25,26^.

This is partly because of the limited dissemination of the plan and the absence of AMR-related courses in many technical training programmes. According to the study, in Benin, AMR education generally begins at the university level and is restricted to specific disciplines related to human health (medicine, pharmacy, nursing, obstetrics and laboratory sciences). Workers in environmental health, agriculture and food production sectors have received little or no AMR-related education during their training.

Similar observations in other African countries^26^ have led WHO Africa to recommend expansion of education and training to all relevant sectors to promote One Health practices in AMR control^20^. To improve public awareness and education in the fight against AMR, Benin should develop and implement a communication strategy with targeted messages tailored to dissemination channels, ensuring the involvement of all stakeholders, including those in the animal health, agriculture and food production sectors. It is imperative to strengthen and integrate training modules on AMR tailored to the specific needs of the different stakeholders.

### Surveillance, laboratory capacity and antimicrobial resistance research

The study revealed limitations in Benin’s capacity for effective AMR surveillance at the national level although there are around 20 bacteriology laboratories capable of generating antibiotic susceptibility data across different sectors. According to 2023 statistics from the DGMHED, the main AMR-associated pathogens in Benin are *Escherichia coli, Staphylococcus aureus, Klebsiella pneumoniae, Streptococcus pneumoniae, Acinetobacter baumannii and Pseudomonas aeruginosa*, which is consistent with reports from some others countries^3^.

Antimicrobial resistance surveillance is essential for tracking resistance trends, identifying emerging threats and informing public health decision-making^1,20^. To this end, Benin should invest in bacteriology laboratory equipment, the implementation of data management systems and the strengthening of staff training. It is necessary to improve coordination between laboratories in different sectors to ensure an effective exchange of information and a harmonised approach to AMR surveillance. A designated reference laboratory equipped with modern devices should conduct external quality assessments for network laboratories while helping them to confirm cases of multidrug-resistant bacteria (MDR)^15^.

Antimicrobial resistance surveillance must go hand in hand with monitoring antimicrobial consumption and use, as inappropriate usage drives AMR emergence and spread^24,25^. Benin has yet to establish such a system, and monitoring antimicrobial consumption remains difficult, especially in animal health and agriculture sectors. These challenges are similar in many West African countries, where unregulated antimicrobials frequently cross borders^7,13^.The lack of surveillance of antibiotic prescriptions and rational use was proven in a study conducted in northern Benin, which revealed that 58.24% of antibiotic prescriptions were unjustified, 14.36% were inappropriate for the condition and 7.18% had incorrect dosages^27^. Another study showed that only 22.7% of antibiotic prescriptions for hospitalised patients were based on an antibiogram examination^28^. It is urgent to finalise and validate the guidelines on AMR surveillance, antimicrobial consumption (AMC) and antimicrobial use (AMU)^1^. The objectives regarding the WHO AWaRe classification of antibiotics^28^ must be applied in the country.

### Infection prevention and control

Infection prevention and control measures are critical for preventing the spread of AMR. Despite the adoption of a 5-year IPC national plan in 2024, Benin has yet to establish a national healthcare-associated infection control programme. A well-funded national programme with trained personnel, aligned with WHO recommendations, would facilitate the adoption of standardised IPC guidelines^29^. These guidelines, based on international standards while considering local realities, would help healthcare facilities improve hygiene practices such as hand hygiene, environmental cleaning, sterilisation, hospital linen management, waste disposal and surveillance of healthcare-associated infections^29,30,31^. Implementing water, sanitation and hygiene (WASH) programmes in healthcare settings is also crucial for IPC efforts^32^.

Promoting IPC and WASH in community settings is equally essential for AMR reduction. Particular attention should be given to animal health facilities, including veterinary clinics, livestock farms, aquaculture units, agricultural enterprises and food production and distribution units^17,32,33^. Currently, Benin lacks national guidelines to harmonise hygiene practices across these critical sectors. In the meantime, awareness campaigns should be conducted to educate veterinarians, para-veterinarians, butchers, livestock farmers and food safety inspectors on best hygiene practices^7^.

### Antimicrobial management and use

This study shows that Benin has made efforts to improve quality assurance in the drug supply chain, particularly in human health. In contrast to animal health, where the country relies exclusively on drug approvals from the West African Economic and Monetary Union (WAEMU)^34,35^, medicines for human health are regulated by the Beninese Agency for Medicines and Other Health Products (ABMed). This agency oversees the entire marketing authorisation process in collaboration with the National Agency for Quality Control of Health Products and Water (ANCQ) for pharmaceutical compliance analysis^35,36^.

However, significant efforts are still needed to combat substandard and falsified antimicrobials. Despite these efforts, limited resources to effectively track offenders persist. This challenge is widespread across many West African countries^36,37^. Regulatory enforcement in the agricultural sector remains weaker. It is essential to strengthen these efforts and enforce existing regulations to prevent the circulation of counterfeit medicines on the market^34,35^. Through the One Health platform, coordinated actions should be intensified across all relevant sectors to address the risks associated with substandard or falsified pharmaceutical products. This includes banning the use of antimicrobials as growth promoters^38^, an essential measure to reduce selective pressure favouring resistance. Implementing a clear regulatory framework governing veterinary medicine and phytosanitary products would be impactful. Since 2022, the electronic Logistics Management Information System (eSIGL) has been deployed in health centres and district warehouses. This system offers the possibility to monitor antimicrobial consumption if standardised protocols are widely adopted and stakeholders are adequately trained. This would facilitate stock management while reducing the risks of shortages or expirations of antimicrobials at multiple levels^39^. Benin has initiated the development of a national guide for the appropriate use of antimicrobials, including protocols for antibiotic prophylaxis and empirical therapy, in order to reduce unnecessary prescriptions^7,27^.

### Study limitations

This study has some limitations. Although six departments were selected to provide a representative sample, the results may not fully reflect the situation in all regions of Benin. Data collection relied in part on self-reported information from stakeholders, which may introduce reporting bias. The assessment of implementation levels was based on expert judgement and available documentation, which may involve some subjectivity. Despite these limitations, the use of multiple data sources and the inclusion of different perspectives strengthen the validity of our conclusions.

## Conclusion

The evaluation of Benin’s National Multisectoral AMR Action Plan 2019–2024 reveals both notable progress and significant challenges in the fight against AMR. While efforts such as the development of regulatory frameworks, establishment of AMR focal points and the improvements of antimicrobial quality assurance have been commendable, significant gaps persist. These include the absence of a fully operational MCG with a clear mandate, mobilisation of financial resources, limited surveillance systems and the need for stronger communication and education initiatives in all sectors. Concerns regarding the circulation of substandard and falsified antimicrobials, coupled with limited law enforcement resources, also remain a significant obstacle. For the period 2025–2030, priorities must be establishing and empowering a dedicated coordination group, developing a reference laboratory and a comprehensive monitoring system, adopting standardised IPC and WASH practices, enforcing stricter prescription regulations and banning antimicrobials as growth promoters and mobilising resources through increased multisectoral collaboration.
